# Body Composition in Young Adults Living With a Fontan Circulation: The Myopenic Profile

**DOI:** 10.1161/JAHA.119.015639

**Published:** 2020-04-15

**Authors:** Derek Tran, Paolo D'Ambrosio, Charlotte E. Verrall, Chantal Attard, Julie Briody, Mario D'Souza, Maria Fiatarone Singh, Julian Ayer, Yves d'Udekem, Stephen Twigg, Glen M. Davis, David S. Celermajer, Rachael Cordina

**Affiliations:** ^1^ Sydney School of Health Sciences The University of Sydney Camperdown New South Wales Australia; ^2^ Sydney Medical School The University of Sydney Camperdown New South Wales Australia; ^3^ Department of Cardiology Royal Prince Alfred Hospital Camperdown New South Wales Australia; ^4^ Heart Centre for Children The Children’s Hospital at Westmead New South Wales Australia; ^5^ Haematology Research Group Murdoch Children’s Research Institute Parkville Victoria Australia; ^6^ Department of Nuclear Medicine The Children’s Hospital at Westmead New South Wales Australia; ^7^ Department of Paediatrics Faculty of Medicine The University of Melbourne Parkville Victoria Australia; ^8^ Department of Cardiac Surgery Royal Children’s Hospital Melbourne Parkville Victoria Australia; ^9^ Department of Endocrinology Royal Prince Alfred Hospital Camperdown New South Wales Australia

**Keywords:** congenital heart disease, exercise capacity, muscle wasting, obesity, single ventricle, Congenital Heart Disease

## Abstract

**Background:**

We sought to characterize body composition abnormalities in young patients living with a Fontan circulation and explore potential pathophysiologic associations.

**Methods and Results:**

Twenty‐eight patients with a Fontan circulation were prospectively recruited in this cross‐sectional study. Participants underwent cardiopulmonary exercise testing, dual‐energy X‐ray absorptiometry, echocardiography, and biochemical assessment. Mean age was 26±7 years. Skeletal muscle mass, estimated by appendicular lean mass index Z score, was reduced compared with reference data (−1.49±1.10, *P*<0.001). Percentage body fat Z score overall was within normal range (0.23±1.26, *P*=0.35), although 46% had elevated adiposity. Those with reduced skeletal muscle mass (appendicular lean mass index Z score of −1 or lower) had lower percent predicted oxygen pulse (55±15 versus 76±16%, *P*=0.002). Overall agreement between body mass index and dual‐energy X‐ray absorptiometry to assess adiposity was fair only (weighted [linear] κ coefficient: 0.53; 95% CI, 0.34–0.73) and slight in the setting of muscle mass deficiency (weighted κ coefficient: 0.32; 95% CI, 0.13–0.50). Appendicular lean mass was independently associated with absolute peak VO_2_ (β=70.6 mL/min, *P*=0.001). Appendicular lean mass index Z score was inversely associated with hemoglobin (*r*=−0.4, *P*=0.04), and the degree of muscle deficit was associated with ventricular systolic impairment.

**Conclusions:**

Young patients with a Fontan circulation have a body composition characterized by reduced skeletal muscle mass, which is associated with peak exercise capacity. Increased adiposity is common despite a normal body mass index. Low skeletal muscle mass is associated with systolic dysfunction and compensatory erythrocytosis.

**Clinical Perspective**

**What Is New?**

- We describe a pattern of myopenia in a relatively well cohort living with a Fontan circulation and the “Fontan‐associated myopenia” phenotype.
- Despite normal range body mass index, lean mass deficits conceal the presence of increased adiposity.
- Ventricular systolic function and hemoglobin levels are negatively associated with skeletal muscle mass and may reflect important pathophysiologic contributors.

**What Are the Clinical Implications?**

- Body mass index is an inadequate measure of adiposity and those with normal body mass index frequently have excessive adiposity when assessed with dual‐energy X‐ray absorptiometry.
- Therapies directed toward improving body composition profile may be beneficial in the management of patients with a Fontan circulation who are dependent on the skeletal muscle pump for venous return.

**Nonstandard Abbreviations and Acronyms**

**%BF** Percentage body fat

**ALMI** Appendicular lean mass index

**DXA** Dual‐energy X‐ray absorptiometry

**NYHA** New York Heart Association

**RPAH** Royal Prince Alfred Hospital

**VO_2_** Oxygen uptake

The Fontan procedure^1^ is the surgical pathway of choice for babies born with a functionally single ventricle unsuitable for biventricular repair. The procedure involves redirecting venous return from the superior and inferior vena cavae directly to the pulmonary arteries, bypassing the heart. This results in a unique circulation with no subpulmonary pump and a single functioning systemic ventricle. Because of advances in care, the majority of children palliated with a Fontan circulation now survive to adulthood.^2^ Although prognosis has markedly improved, exercise intolerance, reduced quality of life and long‐term complications associated with multiorgan dysfunction are common.^3^ Chronically elevated central venous pressure, low cardiac output, and cyanosis can contribute to the development of complications including heart failure, arrhythmias, thrombosis, hepatic fibrosis, and protein‐losing enteropathy.

We previously demonstrated that an important deficiency of skeletal muscle mass exists in this population, with significant implications for venous return and exercise capacity.^4^, ^5^ Our understanding of body composition in this unique physiologic setting remains limited. In acquired biventricular heart failure, hormonal imbalance contributes to muscle wasting and dysfunction.^6^ The influence of hormonal profile on lean and fat mass is poorly characterized in the setting of the Fontan circulation, as are other pathophysiologic contributors. Obesity is likely to also have a central influence. Adverse cardiovascular effects of excess adipose tissue are well recognized, and there appears to be a J‐ or U‐shaped relationship with body mass index (BMI) and all‐cause mortality in the general population.^7^ In the setting of congenital heart disease, Brida et al^8^ found an association with higher BMI and lower mortality. Whether these effects are consistent or even more significant in adults with a Fontan circulation remains unclear.

This study aimed to characterize body composition in a cohort of clinically stable adults living with a Fontan circulation. We hypothesized that there would be a predisposition towards unfavorable body composition with reduced skeletal muscle mass and increased adiposity. We also sought to explore possible contributors to altered body composition and the influence of body composition on exercise capacity and muscle function.

## Methods

### Study Population and Design

Twenty‐eight patients (≥16 years) with a Fontan circulation were prospectively recruited from March 2016 to February 2018 from the Adult Congenital Heart Disease Database at Royal Prince Alfred Hospital (RPAH) and the Australian and New Zealand Fontan Registry. Exclusion criteria included New York Heart Association (NYHA) class III or IV, major intellectual or physical disability, and current pregnancy. Informed consent was obtained from all participants. This study was approved by the Sydney Local Health District Ethics Review Committee (HREC/15/RPAH/322). The data for this study may be made available for scientific purposes from the corresponding author on reasonable request.

### Body Composition Analysis

A trained technician performed a total body composition scan by dual‐energy X‐ray absorptiometry (DXA‐Lunar Prodigy [GE Healthcare], enCore v13.6). Analysis of body composition measures was completed by adjusting an automatically generated template according to reference points described by the manufacturer. Values for total lean mass and appendicular lean mass (sum of arms and legs) were obtained. Lean mass indexes were calculated by dividing lean mass by height squared (kg/m^2^) and expressed as Z scores. The appendicular lean mass index (ALMI) Z score was used as a measure for skeletal muscle mass.^9^ Total percentage body fat (%BF) was calculated by dividing total fat mass by total body mass (sum of bone mineral content, lean mass, and fat mass). Fat mass index was calculated by dividing total fat mass by height squared.

ALMI and %BF (adiposity) were converted into Z scores using age‐sex matched reference values.^10^, ^11^ Body composition Z scores for participants aged <20 years were determined using an updated version of previously published normal reference data from the Children's Hospital at Westmead database.^12^ Participants were categorized into normal skeletal muscle mass and skeletal muscle deficit groups using ALMI Z scores. Normal skeletal muscle mass was considered an ALMI Z score higher than −1, and skeletal muscle deficit was defined as an ALMI Z score of −1 or lower. Fontan associated myopenia was defined as patients with significant skeletal muscle deficits in the sarcopenic range (Z score: −2 or lower).

BMI was used as a surrogate for adiposity, and participants were categorized as having low (underweight), normal (normal weight), moderate (overweight), and high (obese) BMI adiposity, using the World Health Organization BMI definitions for adolescents and adults.^13^, ^14^, ^15^ To compare BMI and DXA for assessing adiposity, DXA reference %BF cut‐point values^10^, ^16^ were used to categorize low, normal, moderate, and high adiposity levels; %BF was also calculated relative to ideal reference lean mass^11^ to understand whether it changed the adiposity category that was of particular significance in those with skeletal muscle mass deficiency.

### Blood Biochemistry

To explore any potential biochemical and hormonal abnormalities that may contribute to body composition abnormalities, each participant underwent laboratory studies. Blood was collected and analyzed by trained staff at the RPAH pathology laboratory using standard assays. Distribution scores were calculated from the hospital laboratory reference ranges or kit inserts to account for sex differences. Both aldosterone (pmol/L) and renin activity (fmol/L per second) were excluded from analysis if the participant was prescribed angiotensin‐converting enzyme inhibitors, angiotensin receptor blockers, and/or an aldosterone antagonist (n=8). Sex hormones were not analyzed in women who were on contraceptives (n=2), and thyroid‐stimulating hormone was not analyzed in participants on thyroid hormone replacement (n=1). Glomerular filtration rate was estimated in adults from serum creatinine (μmol/L) using the Chronic Kidney Disease Epidemiology Collaboration formula. We did not calculate glomerular filtration rate for patients aged <18 years (n=4). Vitamin D deficiency was defined if 25‐hydroxy‐vitamin D was <50 nmol/L.

### Cardiopulmonary Exercise Testing and Spirometry

Our methods have been described previously in detail.^17^ In brief, participants performed an individualized ramp protocol on an electronically braked cycle ergometer. Oxygen uptake (VO_2_), carbon dioxide production, ventilation, heart rate, and work rate were simultaneously collected. Oxygen pulse (mL/beat) was calculated by dividing VO_2_ by heart rate. Spirometry was performed before every exercise test. A respiratory scientist determined peak VO_2_ as a 20‐second average and the anaerobic threshold using the V‐slope method. Peak VO_2_ and peak oxygen pulse are expressed as a percentage of predicted values from standard reference equations to account for sex, weight, height, and age differences, as appropriate.^18^, ^19^

### Muscle Function

Handgrip strength (kg) was measured standing at a 90° elbow flexion angle on both hands using a handgrip dynamometer (TKK 5101 Grip‐D; Takei). At least 2 trials were performed on each hand. Maximal handgrip strength was defined as the highest value achieved from either hand and converted to a percentage of normal reference values.^20^

### Echocardiography

Our echocardiographic method has been described elsewhere.^21^ In brief, cardiac function was assessed at the RPAH Department of Cardiology using cardiac ultrasound machines (EPIQ 7C or iE33; Phillips) by trained technicians. Systolic function was qualitatively reported on a categorical scale as normal, mild, moderate, or severe impairment by a cardiologist with expertise in echocardiography and congenital heart disease who was blinded to the body composition results. Ejection fraction estimates were not used because 3‐dimensional volumes were not available in all participants, and Simpson's rule is invalid in the majority of complex univertricular hearts.^22^

### Statistical Analysis

Data are expressed as the mean±SD for normally distributed data or median and interquartile range for nonnormally distributed data unless specified otherwise. Statistical analysis was performed using IBM SPSS v24 software (IBM Corp). The Shapiro–Wilk test was used to assess normal distribution. In this exploratory analysis, to compare differences between patients with normal skeletal muscle mass and skeletal muscle deficits, a Fisher exact test was used for categorical variables, an independent *t* test was used for normally distributed variables, and a Mann–Whitney *U* test was performed for nonnormally distributed variables. To investigate any associations with skeletal muscle mass and adiposity, a Spearman correlation was used given the small sample size and to account for nonnormally distributed data. To analyze both sexes together in the setting of sex‐differentiated values, blood biochemistry was normalized for sex using reference values and converted to a T and/or Z distribution score for correlation analysis. The Mantel–Haenszel test of trend was used to investigate an association between skeletal muscle mass phenotypes (ie, normal skeletal muscle mass, less pronounced skeletal muscle mass deficit, or Fontan‐associated myopenia) and ventricular systolic function. Skeletal muscle phenotypes were categorized as (1) normal skeletal muscle mass, (2) less pronounced skeletal muscle mass deficit, and (3) Fontan‐associated myopenia. Ventricular systolic function was graded as (1) normal, (2) mild impairment, or (3) moderate impairment. Weighted (linear) κ coefficient (k_w_) was used to test the level of agreement for categorizing adiposity using BMI and sex‐age‐specific %BF cut points. Factors associated with exercise capacity were determined using linear regression models. Univariable analysis was performed to explore potential variables associated with exercise capacity. Statistically significant variables from the univariable analyses and factors known to influence exercise capacity (ie, sex, age, and body composition) were selected for the multivariable model to adjust for confounding variables. Statistical significance was accepted as *P*<0.05.

## Results

### Participant Characteristics

Participant characteristics are reported in Table 1. The mean age of our cohort was 26±7 years, 54% of participants were female, and the median BMI was 22.4 kg/m^2^ [interquartile range: 20.4–27.3], implying normal weight overall. Participants with normal muscle mass had higher BMI than those with muscle mass deficits. Systolic function was normal in 79% of the cohort, 57% of participants had a predominant left ventricle, and the most common type of Fontan circulation was the extracardiac conduit (50%). All participants with normal range skeletal muscle mass had normal ventricular systolic function. Five of the 6 participants (80%) with mild or moderate ventricular systolic impairment also had Fontan‐associated myopenia. The Mantel–Haenszel test of trend test showed a linear trend between the degree of skeletal muscle deficit and grade of ventricular systolic impairment (χ^2^[1] =5.7, *P*=0.02, *r*=0.5). Lower muscle mass was associated with worse ventricular systolic function.

**Table 1:** Participant Characteristics

	All Fontan Participants (n=28)	Normal Skeletal Muscle Mass (n=9)	Skeletal Muscle Deficit (n=19)	*P* Value^a^
Sex (female/male), n/n	15/13	7/2	8/11	0.11
Age, y	26±7	24±7	27±6	0.33
BMI, kg/m^2^	22.4 [20.4–27.3]	27.2±5.7	22.1±3.4	0.03
BMI Z score	0.38±1.32	1.32±1.28	−0.06 ±1.11	0.007
Height, m	1.67±0.12	1.65±0.09	1.68±0.12	0.55
Weight, kg	62.7 [54.5–72.3]	68.1 [60.4–91.9]	60.2 [51.2–71.6]	0.09
BSA, m^2^	1.7±0.2	1.8±0.2	1.7±0.2	0.21
Predominant ventricular morphology, n (%)				1.00^b^
Left	16 (57)	6 (67)	10 (53)	
Right	8 (29)	2 (22)	6 (32)	
Univentricle	2 (7)	1 (11)	1 (5)	
Indeterminate	2 (7)	0 (0)	2 (11)	
Type of Fontan, n (%)				0.23^c^
Extracardiac	14 (50)	4 (44)	10 (53)	
Intracardiac	11 (39)	3 (33)	8 (42)	
Atriopulmonary	3 (11)	2 (22)	1 (5)	
Fenestration	9 (32)	3 (33)	6 (32)	1.00
Systolic function, n (%)				1.00^d^
Normal	22 (79)	9 (100)	13 (68)	
Mildly impaired	5 (18)	0 (0)	5 (26)	
Moderately impaired	1 (4)	0 (0)	1 (5)	
Severely impaired	0 (0)	0 (0)	0 (0)	
AV valve regurgitation, n (%)				0.14^e^
None	10 (36)	4 (44)	6 (32)	
Mild	13 (46)	5 (56)	8 (42)	
Moderate	4 (14)	0 (0)	4 (21)	
Severe	1 (4)	0 (0)	1 (5)	
Systemic valve regurgitation, n (%)				1.00^f^
None	23 (82)	9 (100)	14 (74)	
Mild	4 (14)	0 (0)	4 (21)	
Moderate	1 (4)	0 (0)	1 (5)	
Severe	0 (0)	0 (0)	0 (0)	
Medications, n (%)				
Antiplatelets	16 (57)	6 (67)	10 (53)	0.7
Warfarin/DOACs	10 (36)	3 (33)	7 (37)	1.00
ACEI/ARB	6 (21)	2 (22)	4 (21)	1.00
β‐Blocker	2 (7)	1 (11)	1 (5)	1.00
Digoxin	1 (4)	0 (0)	1 (5)	1.00
Diuretics	5 (18)	3 (33)	2 (11)	0.29
Antiarrhythmic	4 (14)	2 (22)	2 (11)	0.57
NYHA class, n (%)				0.65^g^
Class I	21 (75)	6 (67)	15 (79)	
Class II	7 (25)	3 (33)	4 (21)	
Pacemaker	6 (21)	2 (22)	4 (21)	1.00

Data shown are mean*±*SD or median [interquartile range] except as noted. ARB indicates angiotensin II receptor blocker; ACEI, angiotensin‐converting enzyme inhibitor; AV, atrioventricular; BMI, body mass index; BSA, body surface area; DOAC, direct‐acting anticoagulant; and NYHA, New York Heart Association.

a *P* value for normal skeletal muscle mass compared with skeletal muscle mass deficit groups.

b Right ventricle morphology vs left ventricle, univentricular and indeterminate ventricle morphology.

c Extra‐ and intracardiac vs atriopulmonary connection.

d Normal or mildly impaired systolic function vs moderate or severe systolic function.

e No or mild AV valve regurgitation vs moderate or severe AV valve regurgitation.

f No or mild systolic valve regurgitation vs moderate and severe systolic valve regurgitation.

g NYHA class I vs NYHA class II.

### Body Composition

**Figure 1:**
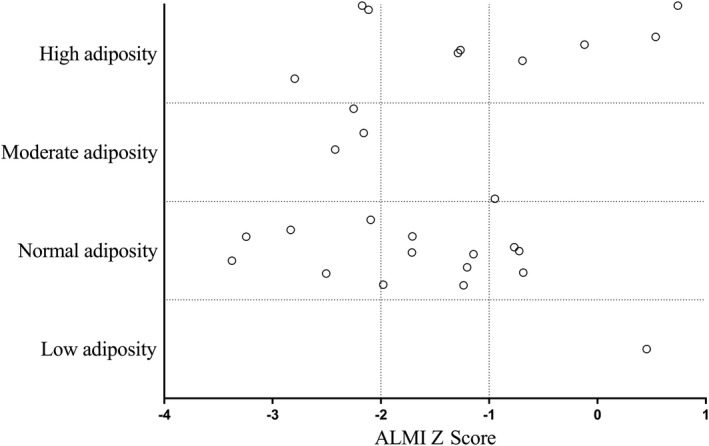
Body composition profiles of all Fontan participants based on dual‐energy X‐ray absorptiometry. The *y*‐axis represents age‐ and sex‐adjusted percentage body fat (adiposity levels) and *x*‐axis appendicular lean mass index (ALMI) Z score (surrogate for skeletal muscle mass). An ALMI Z score of −1 or lower indicates skeletal muscle mass deficit, and −2 or lower is used to define Fontan‐associated myopenia.

Results for body composition are shown in Table 2. Individual body composition profile is shown in Figure 1. Of the 28 participants recruited, 11 (39%) had Fontan‐associated myopenia (Z score: −2 or lower), 8 (29%) had less pronounced skeletal muscle mass deficit (Z score: higher than −2 and −1 or lower), and only 9 (32%) had normal range muscle mass (Z score: higher than −1). The majority of participants with normal muscle mass were female (7 of 9), although there was no association between sex and skeletal muscle deficit (*P*=0.11). There was no difference in ALMI between participants with vitamin D deficiency (n=7) and those who were replete (6.8±1.2 versus 6.1±0.8 kg/m^2^, *P*=0.14).

**Table 2:** Body Composition Results

	All Fontan Participants (n=28)	Normal Skeletal Muscle Mass (n=9)	Skeletal Muscle Mass Deficit (n=19)	*P* Value^a^
Total lean mass, kg	42.9±8.2	44.4±8.8	42.1±8.0	0.49
Total lean mass index, kg/m^2^	15 [13.9–16.2]	16.2±2.0	14.9±1.1	0.09
Total lean mass index Z score	−0.70±1.15	0.55±0.64	−1.29±0.80	<0.001
Appendicular lean mass, kg	18.0±4.3	18.9±4.4	17.4±4.3	0.38
ALMI, kg/m^2^	6.4±1.0	6.9±1.0	6.1±0.9	0.04
ALMI Z score	−1.49±1.10	−0.25±0.66	−2.08±0.68	<0.001
Trunk to appendicular fat ratio	1.3 [1.1–1.8]	1.3 [0.9–1.4]	1.5 [1.1–1.9]	0.22
Fat mass index, kg/m^2^	6.5 [4–9.9]	9.9±5.3	6.3±3.04	0.08
%BF	30±11	35±13	27±10	0.11
%BF Z score	0.23±1.26	−0.04 [−0.9‐1.9]	−0.32 [−0.8‐1.08]	0.66

Data are presented as mean*±*SD or median [interquartile range]. ALMI indicates appendicular lean mass index; %BF, percentage body fat.

a *P* value for normal skeletal muscle mass compared with skeletal muscle mass deficit groups.

Male participants had lower %BF assessed by DXA compared with female participants (24±10% versus 34±10%, *P*=0.02). Based on DXA %BF, of 28 participants, 9 (32%) had high adiposity, 4 (14%) had moderate adiposity, 14 (50%) had normal range adiposity, and 1 (4%) had low adiposity; 3 participants met the criteria for both Fontan‐associated myopenia and high adiposity by DXA. Adiposity categories if %BF was indexed to ideal reference lean mass are shown in Figure S1.

**Figure 2:**
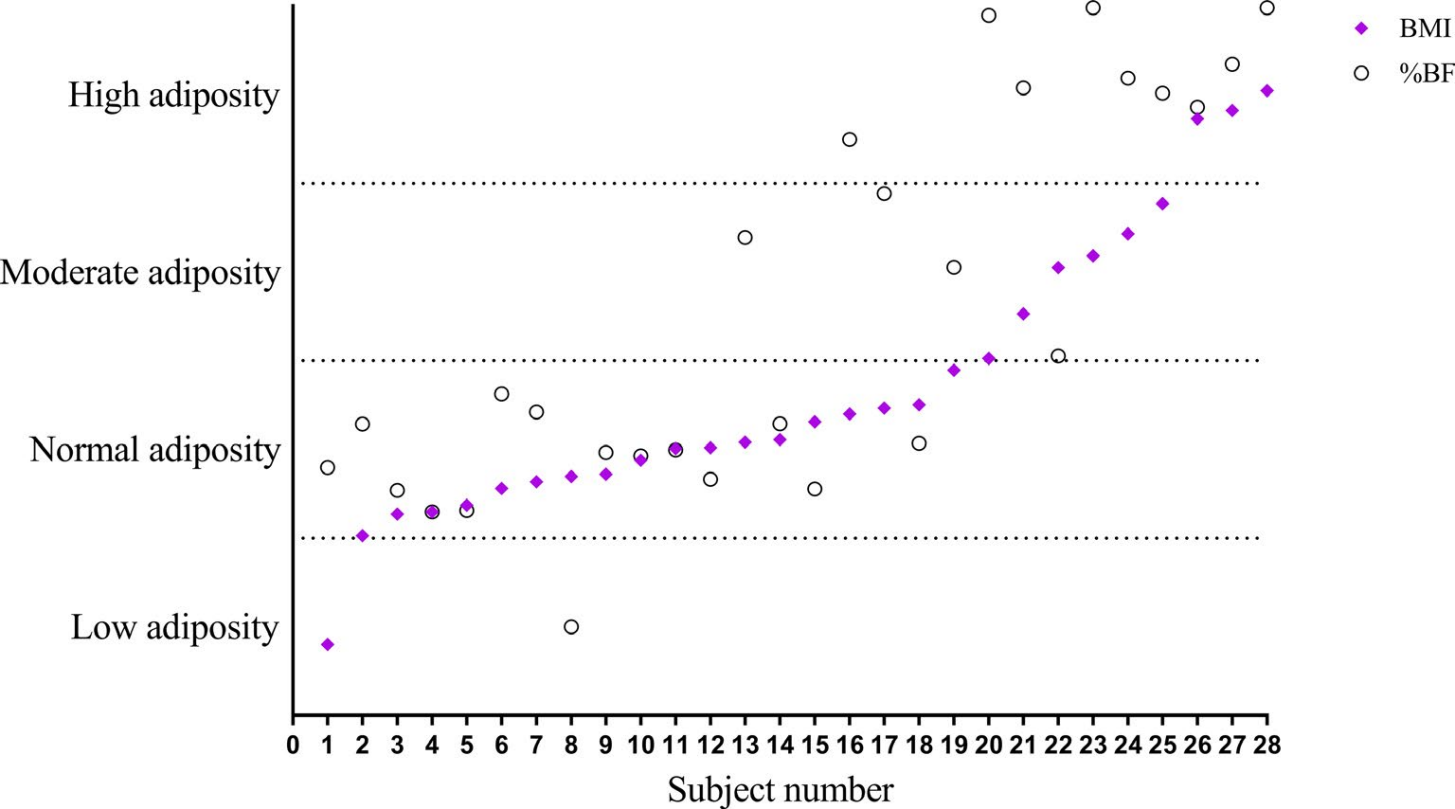
Intrasubject comparison of body mass index (BMI) and percentage body fat (%BF) for categorizing adiposity. Values are expressed as a percentage of the participant's categorical range for both BMI and %BF cut points. There was only 61% agreement between BMI and %BF for categorizing adiposity in patients with a Fontan circulation.

The weighted level of agreement between BMI and DXA by %BF to categorize body composition into low, normal, moderate, or high adiposity was fair only (k_w_=0.53; 95% CI, 0.34–0.73) and is shown in Figure 2. Overall, there was only 61% agreement. In participants with normal muscle mass, the level of agreement was strong (k_w_=0.80; 95% CI, 0.57–1.0), with 78% being placed in the same category. In participants with muscle mass deficits, the level of agreement was relatively weak (k_w_=0.32; 95% CI, 0.13–0.50), and only 53% of participants were classified in the same category.

### Biochemistry

Detailed biochemical results are reported in Table S1. Technical difficulties resulted in missing data for NT‐pro‐BNP (N‐terminal probrain natriuretic peptide; n=3), 25‐hydroxy‐vitamin D (n=2), international normalized ratio (n=2), high‐sensitivity C‐reactive protein (n=1), parathyroid hormone (n=1), and all endocrine biomarkers for 1 participant. Among the hormonal measures, 40% of participants had above‐normal‐range parathyroid hormone, even though only 7 participants had a 25‐hydroxy‐vitamin D level <50 nmol/L. Blood leptin was increased in the majority (70%) of participants, reflecting the elevated adiposity levels across the cohort. There was no difference in NT‐pro‐BNP between normal and skeletal muscle mass deficit groups (22.4 pmol/L [interquartile range: 7.9–56.7] versus 13.1 pmol/L [interquartile range: 6.4–23.3]; *P*=0.5).

### Factors Associated With Exercise Capacity

The univariable and multivariable linear regression results associated with exercise capacity as the dependent variable are shown in Table 3. Both greater appendicular lean mass and sex were associated with peak VO_2_ (mL/min). Appendicular lean mass remained independently associated with peak VO_2_ after including sex, age, and fat mass in the model. For every kilogram increase in appendicular lean mass, peak VO_2_ increased by 70.6 mL/min.

**Table 3:** Univariable and Multivariable Linear Regressions Between Peak VO_2_ (mL/min) and Potential Associated Factors

Variable	Level	Univariable		Multivariable	
		Difference (95% CI)	*P* Value	Difference (95% CI)	*P* value
Age, y		−0.12 (−30.5, 30.3)	0.99	3.0 (−17.7, 23.8)	0.77
Sex	Female	−616.5 (−932.3, −300.7)	<0.001	−235.9 (−610.9, 139)	0.22
	Male	Reference		Reference	
Appendicular lean mass, kg		89.5 (57.4, 121.6)	<0.001	70.6 (26.8, 114.5)	0.002
Total fat mass, kg		3.45 (−14.5, 21.4)	0.71	2.5 (−10.6, 15.7)	0.70
%BF		−9.0 (−26.3, 8.3)	0.31		
Hb, g/L		10.3 (−3.7, 24.2)	0.15		
NYHA class	NYHA class I	−46.3 (−494.8, 402.3)	0.84		
	NYHA class II	Reference			
Systolic function	Normal	51.6 (−997, 1100.3)	0.92		
	Mildly impaired	−51.2 (−1174.65, 1072.3)	0.93		
	Moderately impaired	Reference			
Resting SpO_2_, %		−28.3 (−80.6, 24.0)	0.29		
Peak exercise SpO_2_, %		−25.02 (−55.9, 5.9)	0.11		

%BF indicates percentage body fat; Hb, hemoglobin; NYHA, New York Heart Association; and SpO_2_, oxygen saturation.

### Muscle Function

Peak handgrip strength was 86±20% of reference values. Participants with normal muscle mass had higher maximal handgrip strength compared with participants with muscle deficits (98±19% versus 80±18%, *P*=0.03). Subgroup analysis showed a lower peak handgrip strength in participants with Fontan‐associated myopenia compared with those without (95±19% versus 72±11%, *P*<0.001).

### Cardiopulmonary Exercise Testing and Spirometry

There was no difference in spirometry measures between normal and reduced muscle mass groups. Overall, forced expiratory volume in one second was 79±12% predicted, and forced vital capacity was 79±11% predicted. The forced expiratory volume in one second/forced vital capacity ratio was 86±5%.

Detailed cardiopulmonary exercise testing results are shown in Table 4. The percentage of predicted oxygen pulse was lower in those with low skeletal muscle mass (76±16% versus 55±15%, *P*=0.002). The resting and peak exercise oxygen saturation for 1 participant could not be interpreted and was removed from the analysis. Two participants had a respiratory exchange ratio of <1.05, suggesting submaximal effort; removing them did not affect the comparative *P* values. The anaerobic threshold for 1 participant could not be identified.

**Table 4:** Cardiopulmonary Exercise Testing Results

	n	All Fontan Participants	n	Normal Skeletal Muscle Mass	n	Skeletal Muscle Mass Deficit	*P* Value^a^
Resting SpO_2_, %	27	93 [92–98]	9	95 [90–98]	18	93 [93–97]	0.86
Peak VO_2_, % predicted	28	66±16	9	71±19	19	64±14	0.28
Peak oxygen pulse, % predicted	28	61±18	9	76±16	19	55±15	0.002
Peak exercise SpO_2_, %	27	91±6	9	90±8	18	92±4	0.5
Maximal HR, % predicted	28	79±11	9	77±14	19	80±9	0.61
Peak RER	28	1.2±0.1	9	1.2±0.1	19	1.2±0.1	0.23
VO_2_ at AT, % of predicted VO_2_	27	45±12	9	44±14	18	46±11	0.69

Data are presented as mean±standard deviation or median [interquartile range]. AT indicates anaerobic threshold; HR, heart rate; RER, respiratory exchange ratio; SpO_2_, oxygen saturation; and VO_2_, oxygen uptake.

a *P* value for normal skeletal muscle mass compared with skeletal muscle mass deficit groups.

### Correlation Analysis

Body composition correlations are reported in Table 5. ALMI Z score correlated inversely with hemoglobin and positively with handgrip strength and percentage predicted oxygen pulse. The %BF Z score, as expected, correlated with higher blood leptin and lower adiponectin levels. It also correlated positively on this univariate analysis with higher resting and peak exercise oxygen saturation. There were no correlations between sex hormones and skeletal muscle mass.

**Table 5:** Body Composition Correlations

Variable	*r*	*P* Value
ALMI Z score		
BMI Z score	0.49	0.008
Handgrip strength, % predicted	0.53	0.004
Peak oxygen pulse, % predicted	0.50	0.007
Peak VO_2_, % predicted	0.22	0.25
Hemoglobin, g/L	−0.39	0.04
%BF	0.31	0.11
Total fat mass, kg	0.40	0.04
Fat mass index, kg/m^2^	0.36	0.06
Appendicular fat mass index, kg/m^2^	0.44	0.02
%BF Z score		
BMI Z score	0.72	<0.001
Resting SpO_2_, %	0.46	0.02
Peak exercise SpO_2_, %	0.39	0.04
hsCRP, mg/L	0.49	0.01
ALMI, kg/m^2^	0.31	0.11
Leptin T score	0.87	<0.001
Adiponectin, μg/mL	−0.39	0.04

ALMI indicates appendicular lean mass index; BMI, body mass index; %BF, percentage body fat; hsCRP, high‐sensitivity C‐reactive protein; and SpO_2_, oxygen saturation.

## Discussion

To our knowledge, this study is the first to undertake a detailed characterization of body composition, including fat and skeletal muscle mass, using DXA, specifically, in adolescent and adult Fontan patients. In this cohort of clinically stable patients with a Fontan circulation, we found an unfavorable body composition profile, characterized by skeletal muscle mass deficits and a predisposition to adiposity, which is underestimated by BMI and underappreciated in clinical practice. Low skeletal muscle mass was associated with reduced exercise capacity and oxygen pulse (a surrogate for stroke volume), confirming our previous findings. In this study, skeletal muscle mass was positively associated with ventricular systolic function and negatively associated with compensatory erythrocytosis, reflecting the degree of cyanosis.

### Lean Mass Deficit

We observed skeletal muscle mass deficits in more than two‐thirds of the group; 39% were in the sarcopenic range. Skeletal muscle deficiencies in patients with a Fontan circulation are likely caused by the combination of wasting and underdevelopment. Therefore, the term *sarcopenia* is inappropriate. Instead, we propose the term *Fontan‐associated myopenia* to describe significant muscle deficits (Z score: −2 or lower). Our cohort had moderately reduced exercise capacity, in keeping with the current literature,^23^ that was closely associated with lean mass; appendicular lean mass was independently associated with absolute peak VO_2_. The relationship between peripheral muscle mass and exercise capacity has previously been attributed to the effects of the peripheral muscle pump on ventricular filling in the setting of the Fontan circulation that functions without a subpulmonary cardiac pump.^4^, ^24^, ^25^, ^26^ Supporting this notion and consistent with previous findings,^4^ low skeletal muscle mass was associated with reduced oxygen pulse, a surrogate for stroke volume measured during cardiopulmonary exercise testing, although, of note, oxygen pulse also reflects muscle oxidative capacity that is also probably impaired in this setting. We found that grip strength was positively associated with muscle mass and was lower in patients with Fontan‐associated myopenia. This impairment in strength likely relates to decreased muscle cross‐sectional area, intrinsic metabolic muscle abnormalities,^4^ and hypoactivity.

The presence of a fenestration or pulmonary venous collaterals is common in the setting of a Fontan circulation and can cause cyanosis. We did not find a correlation between resting or peak exercise pulse oximetry measures and muscle mass. However, skeletal muscle mass inversely correlated with hemoglobin, which rises in the setting of reduced oxygen saturations (compensatory erythrocytosis) and might reflect the degree of desaturation more accurately than a single saturation measurement. This association warrants further investigation in a larger cohort because hemoglobin levels are also affected by sex, and there tended to be a higher proportion of male than female participants with reduced muscle mass in this current cohort.

Interestingly, we found no association between sex hormones, markers of neurohormonal activation or inflammatory mediators, and skeletal muscle mass, suggesting that anabolic hormones are not an important pathway in muscle wasting. This finding is consistent with previous findings that were unable to detect a relationship between IGF‐1 (insulin‐like growth factor 1) measures and lean mass.^24^ Recently, vitamin D deficiency was shown to be associated with leg lean mass, suggesting a possible mechanistic link.^24^ We found no relationship between vitamin D and lean mass even though our population tended to have elevated parathyroid hormone, likely reflecting subclinical vitamin D deficiency.

Systolic ventricular impairment was associated with low muscle mass, which is consistent with the findings of Turquetto et al,^25^ who found that stroke volume was independently associated with thigh cross‐sectional area. Several potential mechanisms might underlie this relationship. Muscle deficits may at least partially be due to low cardiac output with secondarily reduced skeletal muscle blood flow and delivery of nutrients and plasma anabolic hormones. In the Fontan setting, cardiac output is typically reduced,^27^ with a markedly attenuated increase during exercise.^17^ Superimposed ventricular dysfunction further insults cardiac output and Fontan physiology. A contrasting hypothesis is that ventricular systolic impairment may also be a consequence preload deprivation^28^ exacerbated by a reduced skeletal muscle pump.

### Obesity and Adiposity

We found that adiposity is underestimated when BMI is used to characterize body composition. The inherent limitation of BMI is its inability to distinguish between the contributions of fat and lean mass. We have demonstrated in our Fontan population that increased adiposity can be concealed in the setting of reduced skeletal muscle, which itself is denser than fat. The mean BMI was 23.7 kg/m^2^ (BMI Z score: 0.38±1.32), indicating normal weight overall; however, based on DXA body composition, 46% of patients had excessive adiposity, and almost a third had high adiposity levels. In this relatively young and well cohort, 11% had both Fontan‐associated myopenia and high adiposity when assessed with DXA.

The interaction of adiposity and Fontan physiology is not well characterized. Higher %BF in pediatric patients is associated with a negative trajectory of exercise capacity that can predict future disadvantageous Fontan pathophysiology.^29^ Anecdotally, we have observed important circulatory demise in patients with marked adiposity and surmised that this is related, at least in part, to the impact on the thoracic bellows, which are essential for venous return; however, this hypothesis remains unproven. We did not identify any obvious relationship between cardiopulmonary exercise testing indexes and adiposity, but this result may relate to our small sample size. One study has reported that obese Fontan patients have increased risk of mortality and heart failure,^30^ with those data based on BMI. In a larger series, Chung et al^31^ contradicted this finding and found a lower risk of heart failure in overweight or obese patients.

Importantly, we found a positive correlation between skeletal muscle mass and total fat mass. Compensatory increases in skeletal muscle mass to account for the loading of excess adiposity or better caloric intake could explain this association, but the relationship is likely to be more complex. Patients who are better nourished and/or less sick may have superior skeletal muscle mass and a degree of increased adiposity. The point at which increased adiposity becomes detrimental deserves further investigation. Indeed, it may be that relatively increased adiposity with reduced skeletal muscle mass is an especially pathogenic combination. We found an inverse correlation between adiponectin and %BF. Adiponectin is an insulin sensitizer that has endothelial protective properties. Increasing adiposity in these patients may result in further endothelial dysfunction and hemodynamic deterioration. The elevated leptin levels detected in our cohort likely largely reflects the greater adiposity observed,^32^ and an associated reduction in circulating adiponectin can potentially reflect insulin resistance.^33^

### Clinical Implications

Our findings suggest that young patients living with a Fontan circulation are predisposed to an unfavorable body composition profile characterized by reduced skeletal muscle mass and increased adiposity. Frequent participation in moderate to vigorous physical activity has been associated with superior peak exercise capacity in Fontan children and adults.^34^ Moreover, increasing muscle mass through exercise in the setting of a Fontan circulation has been shown to improve exercise capacity and cardiac output,^17^, ^35^ likely due to enhanced peripheral muscle pump function to augment venous return.^35^ Although increased adiposity has been associated with declining exercise capacity,^29^ the degree to which adiposity is detrimental is unclear; extreme levels are likely to have adverse consequences.

## Limitations

This preliminary, cross‐sectional study design could demonstrate only association and not causation. Our study is limited in participant numbers; therefore, we may have been underpowered to detect some important relationships (type II error). We did not assess current physical activity levels. Differences in muscle mass may simply be attributed to reduced participation in physical activity in patients who are sicker, although all participants in this study were NYHA class I or II. In the setting of lean mass deficits, %BF can overestimate adiposity. Fat mass index evaluates adiposity independent of confounding factors, including lean mass and bone mineral content.^36^ To our knowledge, there is currently a lack of consensus regarding appropriate fat mass index cut points associated with BMI in adolescents and proposed adult cut points were established based on different DXA apparatus and software.^36^ Therefore, we could not stratify adiposity levels using fat mass index. To address this issue and to help us understand whether we might be overestimating adiposity, we also categorized %BF assuming a reference lean mass that shifted only 2 subjects from increased to normal adiposity. We have not adjusted for the compensatory increase in lean mass associated with elevated adiposity levels (lean mass relative to fat mass)^37^ given the absence of local predictive regression reference equations. Consequently, we may be underestimating the degree of skeletal muscle deficit in our cohort. We did not recruit a healthy control reference group, and our analysis was based on published reference values. However, we carefully selected reference values that were obtained from a large local sample population.

## Conclusions

Adolescents and adults with a Fontan circulation have a body composition profile characterized by low skeletal muscle mass and increased adiposity. Adiposity is often clinically underestimated by BMI in patients with lean mass deficits, which are more accurately identified with DXA. The implications of excess adiposity remain unclear. Greater lean mass is associated with superior exercise performance, and interventions to address this deficiency are paramount.

## Sources of Funding

This work was supported by a National Health and Medical Research Council project grant 1065794, “Functional Outcomes After Fontan Surgery,” and National Health and Medical Research Council partnership grant 1076849, “Giving an Adult Life After Fontan Surgery to Those With the Most Severe Congenital Heart Conditions.”

## Disclosures

Professor d'Udekem is a consultant for MSD and Actelion. Dr Cordina is a consultant for Actelion. The remaining authors have no disclosures to report.

## Supporting information

Table S1
Figure S1
