# Supplementary material for: Body Composition in Young Adults Living With a Fontan Circulation: The Myopenic Profile
Source: J Am Heart Assoc. 2020 Apr 15;9(8):e015639. doi: 10.1161/JAHA.119.015639 (PMC7428525; doi:10.1161/JAHA.119.015639)
Supplement: Supplementary file 1 — Table S1 Figure S1 [file JAH3-9-e015639-s001.pdf]

SUPPLEMENTAL MATERIAL

Table S1. Biochemistry results.

Biochemical Result	n	Mean ± SD or Median [IQR]	Lab Reference Range	Above range/below range
**Hb (g/L)**				
**Male**	13	162.4 ± 9.7	130 - 170	2 (15)/0 (0)
**Female**	15	147.5 ± 13	120 - 150	5 (33)/1 (7)
**WCC (×10^9/L)**	28	6.6 ± 2.3	4 - 10	2 (7)/2 (7)
**Plt (×10^9/L)**	28	160 [128.3 – 217.3]	150 - 400	0 (0)/12 (43)
**eGFR (ml/min/1.73m^2)**	24	115.5 [92.5 – 124]	> 60	-/0 (0)
**Albumin (g/L)**	28	48 ± 3.2	38 - 48	12 (43)/0 (0)
**NT-proBNP (pmol/L)**	25	14.1 [7.2 – 29.6]	< 13	13 (52)/-
**INR**	26	1.2 [1.1 – 1.8]	0.9 - 1.2	9 (35)/0 (0)
**25(OH)-D (nmol/L)**	26	66 ± 26.2	> 50	-/7 (27)
**PTH (pmol/L)**	25	5 [3.9 – 8.4]	2 - 6	10 (40)/0
**hsCRP (mg/L)**	27	1.5 [0.6 – 3.1]	< 5.0	2 (7)/-
**TSH (mIU/L)**	26	3 [2 – 5.2]	0.27 - 4.2	8 (31)/0
**IGF-1 (nmol/L)**				
**Male**	13	26.9 ± 11.9	14.3 - 39.2	2 (15)/2 (15)
**Female**	14	27.1 [22.4 – 38.7]	11.8 - 38.6	3 (21)/0 (0)
**Insulin (pmol/L)**	27	47 [34 – 72]	10 - 96	4 (15)/0 (0)
**C peptide (pmol/L)**	27	854 [682 – 1092]	200 - 1200	4 (15)/0 (0)
**Leptin (ng/mL)**				
**Male**	13	11 ± 8.1	2 - 5.6	9 (69)/1 (8)
**Female**	14	21.7 [10.3 – 34]	3.7 - 11.1	10 (71)/0 (0)
**Adiponectin (ug/mL)**	27	18.7 ± 6.8	3 - 30	3 (11)/0 (0)
**SHBG (nmol/L)**				
**Male**	13	41.9 [29.7 – 49.2]	14 - 75	1 (8)/0 (0)
**Female**	12	92 ± 39.1	19 - 120	2 (17)/0
**Testosterone (nmol/L)**				
**Male**	13	16.2 ± 4.2	10 - 30	0 (0)/1 (8)
**Female**	12	0.4 [0.3 – 0.8]	0 - 1.8	0 (0)/0 (0)
**Oestradiol (pmol/L)**				
**Male**	13	102.6 ± 36	< 220	0 (0)/-
**Female**	12	224.5 [164 – 401.5]	< 700	1 (8)/-
**FSH (IU/L)**				
**Male**	13	4.4 ± 2.1	1.5 - 12.4	0 (0)/0 (0)
**Female**	12	6.6 [4.1 – 12.2]	3.5 - 12.5	3 (25)/1 (8)
**LH (IU/L)**				
**Male**	13	5.8 ± 2.1	1.7 - 8.6	2 (15)/0 (0)
**Female**	12	7.4 [4.6 – 11.9]	2.4 - 12.6	2 (17)/1 (8)
**Prolactin (ng/mL)**				
**Male**	13	9 ± 4.2	2 - 16	0 (0)/0 (0)

**Female**	12	9 ± 4.5	2 - 20	0 (0)/0 (0)
**Renin activity (fmol/L/sec)**	19	270 [90 – 709]	130 - 2350	0 (0)/5 (26)
**Aldosterone (pmol/L)**	19	453 [212 – 919]	60 - 980	3 (16)/0 (0)
**DHEAS (μmol/L)**				
**Male**	13	3.9 ± 1.5	3 - 18	0 (0)/3 (23)
**Female**	12	2.8 ± 1.6	2 - 14	0 (0)/3 (25)

Data shown are mean ± standard deviation, median [interquartile range (IQR)] or n (%). 25(OH)-D; 25-hydroxy-vitamin D, DHEAS; dehydroepiandrosterone sulphate, eGFR; estimated glomerular filtration rate, FSH; follicle-stimulating hormone, Hb; haemoglobin, hsCRP; high sensitive C-reactive protein, IGF-1; insulin-like growth factor 1, INR; international normalised ratio, LH; luteinizing hormone, NT-proBNP; N-terminal pro-brain natriuretic peptide, Plt; platelets, PTH; parathyroid hormone, SHBG; sex hormone binding globulin, TSH; thyroid-stimulating hormone, WCC; white cell count.

Figure S1. Intrasubject comparison of percent body fat (%BF) in the setting of actual lean mass and ideal lean mass based on dual energy X-ray absorptiometry.

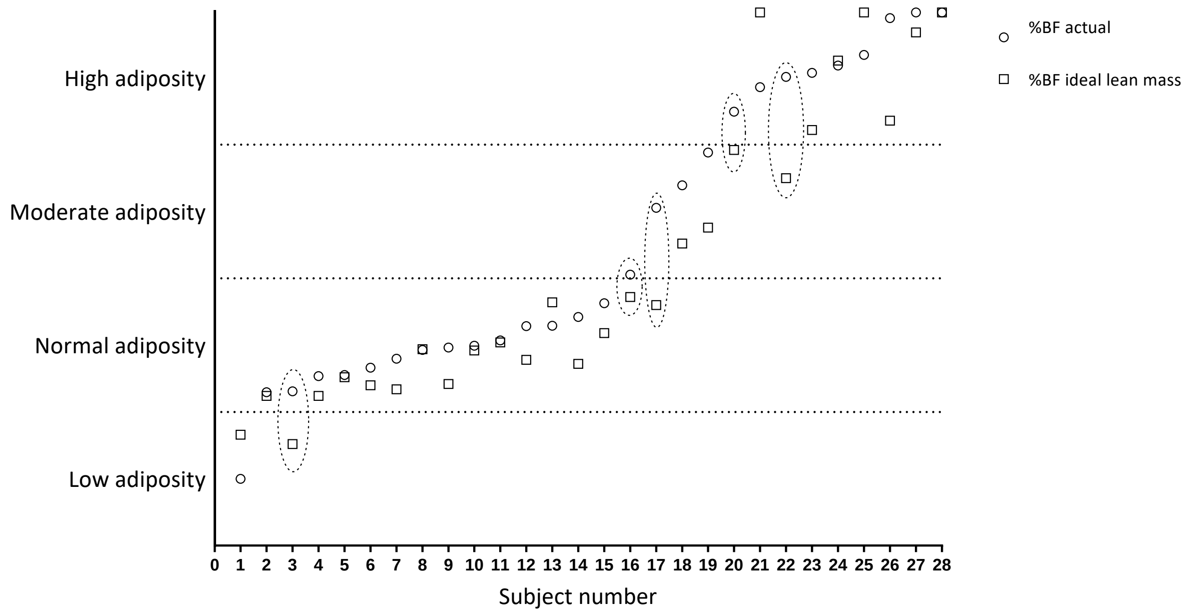

Values are presented as a percentage of each subject's categorical range based on reference cut points. There was 82% agreement despite the potential confounding effect of reduced lean mass in determining adiposity levels.
